# Temperature drives Zika virus transmission: evidence from empirical and mathematical models

**DOI:** 10.1098/rspb.2018.0795

**Published:** 2018-08-15

**Authors:** Blanka Tesla, Leah R. Demakovsky, Erin A. Mordecai, Sadie J. Ryan, Matthew H. Bonds, Calistus N. Ngonghala, Melinda A. Brindley, Courtney C. Murdock

**Affiliations:** 1 Department of Infectious Diseases, College of Veterinary Medicine, University of Georgia, Athens, GA, USA; 2 Center for Tropical and Emerging Global Diseases, University of Georgia, Athens, GA, USA; 3 Department of Population Health, University of Georgia, Athens, GA, USA; 4 Center for Vaccines and Immunology, University of Georgia, Athens, GA, USA; 5 Odum School of Ecology, University of Georgia, Athens, GA, USA; 6 Center of Ecology of Infectious Diseases, University of Georgia, Athens, GA, USA; 7 River Basin Center, University of Georgia, Athens, GA, USA; 8 Biology Department, Stanford University, Stanford, CA, USA; 9 Quantitative Disease Ecology and Conservation Laboratory, Department of Geography, University of Florida, Gainesville, FL, USA; 10 Emerging Pathogens Institute, University of Florida, Gainesville, FL, USA; 11 Department of Mathematics, University of Florida, Gainesville, FL, USA; 12 College of Life Sciences, University of KwaZulu-Natal, Durban, South Africa; 13 Department of Global Health and Social Medicine, Harvard Medical School, Boston, MA, USA

**Keywords:** mosquito, arbovirus, temperature, *Aedes aegypti*, Zika

## Abstract

Temperature is a strong driver of vector-borne disease transmission. Yet, for emerging arboviruses we lack fundamental knowledge on the relationship between transmission and temperature. Current models rely on the untested assumption that Zika virus responds similarly to dengue virus, potentially limiting our ability to accurately predict the spread of Zika. We conducted experiments to estimate the thermal performance of Zika virus (ZIKV) in field-derived *Aedes aegypti* across eight constant temperatures. We observed strong, unimodal effects of temperature on vector competence, extrinsic incubation period and mosquito survival. We used thermal responses of these traits to update an existing temperature-dependent model to infer temperature effects on ZIKV transmission. ZIKV transmission was optimized at 29°C, and had a thermal range of 22.7°C–34.7°C. Thus, as temperatures move towards the predicted thermal optimum (29°C) owing to climate change, urbanization or seasonality, Zika could expand north and into longer seasons. By contrast, areas that are near the thermal optimum were predicted to experience a decrease in overall environmental suitability. We also demonstrate that the predicted thermal minimum for Zika transmission is 5°C warmer than that of dengue, and current global estimates on the environmental suitability for Zika are greatly over-predicting its possible range.

## 1. Introduction

Mosquito-borne viruses are an emerging threat impacting human health and well-being. Epidemics of dengue (DENV), chikungunya (CHIKV) and Zika (ZIKV) have spilled out of Africa to spread explosively throughout the world creating public health crises. Worldwide, an estimated 3.9 billion people living within 120 countries are at risk [1]. In 2015–2016, ZIKV spread throughout the Americas including the continental United States (US), resulting in over 360 000 suspected cases, with probably many more undetected [2]. With the rise of neurological disorders and birth defects, such as Guillain–Barré and congenital Zika virus syndrome [3,4], ZIKV became widely feared and was declared a ‘public health emergency of international concern’ by the World Health Organization in 2016 [5]. In spite of growing research efforts to develop new therapeutics, vaccines and innovative mosquito control technologies, mitigating arbovirus disease spread still depends on conventional mosquito control methods and public education. Thus, substantial efforts have been made to predict how ZIKV will spread seasonally, geographically, and with the effects of climate change (e.g. [6–9]).

There are several key gaps that potentially affect our ability to predict, and ultimately, mitigate the factors that influence transmission risk and arbovirus emergence globally. First, current models predicting mosquito distributions or virus transmission are often limited by a relatively poor understanding of the relationships among mosquito vectors, pathogens and the environment. There is substantial evidence that temperature variability is a key driver of disease transmission across diverse vector-borne pathogen systems (e.g. [8,10,11]). Mosquitoes are small ectothermic animals and their physiology [12,13], life history [8,14] and vectorial capacity [10,15,16] exhibit unimodal responses to changes in temperature. Transmission depends in large part on the ability of mosquitoes to survive the extrinsic incubation period (EIP), become infectious, and bite new hosts, so differential (unimodal) impacts of temperature on survival, vector competence, and EIP have highly nonlinear effects on transmission. Warmer temperatures do not necessarily translate into more infectious mosquitoes [8,17]. Second, current models often ignore the low quality and quantity of existing data. Even in systems that are fairly well-studied (e.g. *Plasmodium falciparum* and DENV), key parameters are often estimated from only a few studies. Finally, current transmission models often assume, with little justification, that the relationship between temperature and EIP is monotonic [18], or that the relationships between temperature, EIP, and vector competence of less-studied arboviruses (e.g. CHIKV and ZIKV) are similar to DENV [8,9,19,20].

To advance our fundamental scientific understanding of the relationship between temperature and ZIKV transmission, we conducted a series of laboratory experiments to estimate the thermal performance of ZIKV (vector competence, the extrinsic incubation rate, and the daily *per capita* mosquito mortality rate) in field-derived *Aedes aegypti* across eight different constant temperatures ranging from 16°C to 38°C. We fit a series of nonlinear functions to estimate the thermal responses of the above traits. These thermal responses were incorporated into a temperature-dependent basic reproductive number (*R*_0_) model developed for *Ae. aegypti* and DENV [14] to infer how temperature variation will impact ZIKV transmission.

## 2. Methods

### (a) Experimental mosquito infections and forced salivations

For details on virus culture and mosquito rearing, see the electronic supplementary material, Methods and Results. For each biological replicate, we separated 8000 1 to 3-day-old females (field derived *Ae. aegypti,* F_4_ generation) and held them for 48 h prior to ZIKV infection (electronic supplementary material, figure S1). Mosquitoes were kept in 1.8 l paper cups and provided with water, which was withdrawn 12 h before feeding. We offered 3–5 day old mosquitoes either an infectious blood meal containing ZIKV at a final concentration of 10^6^ plaque forming units (PFU) ml^−1^ or an uninfected, control blood meal. The blood meal was prepared by washing human blood three times in Roswell Park Memorial Institute medium and the pelleted red blood cells (50%) were resuspended in 33% Delbecco's modified Eagle medium (DMEM), 20% fetal bovine serum (FBS), 1% sucrose, and 5 mmol l^−1^ ATP. For the infectious blood meal, we mixed the blood mixture with ZIKV diluted in DMEM (2 × 10^6^ PFU ml^−1^) at a 1 : 1 ratio. Mosquitoes were blood-fed through a water-jacketed membrane feeder for 30 min, after which we randomly distributed 2000 ZIKV-exposed engorged mosquitoes and 2000 unexposed blood-fed control mosquitoes into mesh-covered paper cups (250 mosquitoes per cup). We then placed one ZIKV-exposed and one control cup at each temperature treatment (Percival Scientific): 16°C, 20°C, 24°C, 28°C, 32°C, 34°C, 36°C and 38°C ± 0.5°C. Chambers were set to 80% ± 5% relative humidity and a 12 : 12 light : dark cycle, and mosquitoes were maintained on 10% sucrose for the duration of the experiment. Mosquito mortality was monitored and recorded daily.

Every three days (up to day 21) we force-salivated 20 ZIKV-exposed mosquitoes per treatment group by immobilizing mosquitoes on ice, removing their legs and wings, and placing the proboscis of each mosquito into a pipet tip (containing 35 µl FBS with 3 mmol l^−1^ ATP) for 30 min on a 35°C warming plate. After salivation, we collected mosquito saliva, heads and legs, and bodies into 700 µl of DMEM with 1× antibiotic/antimycotic. Each tissue was homogenized in a QIAGEN TissueLyzer at 30 cycles s^−1^ for 30 s, and centrifuged at 17 000*g* for 5 min at 4°C. To measure the proportion of mosquitoes that became infected, disseminated infection, and became infectious at each temperature, we tested for the presence/absence of ZIKV in mosquito bodies, legs and heads, and saliva, respectively, using plaque assays on Vero cells (Methods and Results in the electronic supplementary material). Two full biological replicates were performed (electronic supplementary material, figure S1).

### (b) Statistical analysis

Generalized linear model (GLMM) analysis was used to estimate the effects of temperature (*T*; 16°C, 20°C, 24°C, 28°C, 32°C, 34°C, 36°C, 38°C) and days post infection (dpi; 3, 6, 9, 12, 15, 18, 21) on the probability of successful mosquito infection (proportion of mosquitoes with positive bodies), dissemination (proportion of mosquitoes with positive legs and heads), and becoming infectious (proportion of mosquitoes with positive saliva) after being exposed to a ZIKV infectious blood meal. We also used GLMM analysis to estimate the probability of becoming infectious after successful ZIKV infection (proportion of mosquitoes with positive bodies) as a measure of dissemination efficiency. As our response variables were presence or absence of virus in a particular tissue, we constructed our GLMM using a binomial distribution and logit link function. The covariates temperature and dpi were centred by subtracting the mean and scaled by dividing by the standard deviation (SD). To account for differences in ZIKV infection metrics owing to mosquito cohort, we used a random intercept for mosquito cohort in each analysis. Because a diversity of organismal traits exhibit nonlinear, unimodal relationships with temperature [8,21], and we observe non-monotonic effects of dpi on some of our response variables in specific temperature treatments, we incorporated a polynomial function into our statistical model to accommodate this non-linearity. We evaluated a series of eight candidate models which varied in fixed effects structure from a ‘base model’ with only linear fixed effects of temperature and dpi to a ‘full’ model, which included temperature and dpi polynomial terms that were squared (*T*^2^ and *dpi*^2^) and their interactions (R Core Team, 2018 [22], package lme4 [23]). We selected the most parsimonious model using the Akaike information criterion with a sample size correction (AICc). Finally, to estimate the effects of temperature, ZIKV exposure, and their interaction on the daily probability of mosquito survival, we used a Cox proportional hazards model (SAS^®^ Studio, 3.6 Basic Edition) with temperature, infection status (ZIKV-exposed or control), and their interaction as fixed factors, with mosquito batch as a random factor.

### (c) Mechanistic *R*_0_ model

In previous work, we assembled trait thermal response estimates from laboratory experiments that manipulated temperature and measured each of the following traits for *Ae. aegypti* and DENV: egg-to-adult development rate (MDR), survival probability (*p*_EA_), fecundity (EFD; eggs per female per day), biting rate (*a*), adult mosquito mortality rate (*μ*), extrinsic incubation rate (EIR), and vector competence (*bc*; equal to the proportion of exposed mosquitoes that become infected times the proportion of infected mosquitoes that become infectious, with virus in their saliva). We then synthesized them into an estimate for the thermal response of *R*_0_, the expected number of new cases generated by a single infectious person or mosquito introduced into a fully susceptible population throughout the period within which the person or mosquito is infectious [8]:
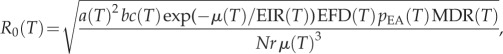
where *N* is the density of humans, *r* is the human recovery rate and (*T*) indicates parameters that are dependent on environmental temperature, *T*. Here, we update three of these thermal response functions—average adult mosquito lifespan (*lf* *=* *1/μ*), EIR and *bc*—using the new experimental data from *Ae. aegypti* mosquitoes exposed to ZIKV-infected blood meals across a range of constant temperatures (see Methods and Results in the electronic supplementary material).

This expression for temperature-dependent *R*_0_ assumes a constant temperature to calculate the per-generation rate of increase of a pathogen in a fully susceptible population. However, environmental temperatures in nature are variable, which affects the calculation and interpretation of *R*_0_ [24–28]. Here, as in previous work [8,15], we use relative *R*_0_ as a simple metric for the *relative* suitability of temperature for transmission, rather than as an absolute metric for secondary case distributions, invasion and extinction thresholds, or expected equilibrium prevalence [29–31]. The relative *R*_0_ approach allows us to estimate the thermal optimum and limits, at which *R*_0_ is maximized or goes to zero, respectively, and compare them to a similar model previously parametrized for DENV [8]. Because our estimate of *R*_0_(*T*) is relative (rescaled to range from zero to one), we cannot estimate the stable transmission threshold *R*_0_(*T*) > 1, so we instead use the more conservative suitability threshold *R*_0_(*T*) > 0. At temperatures outside of this suitable range transmission is impossible because one or more processes necessary for transmission has gone to zero.

### (d) Mapping seasonal transmission range

To illustrate predicted temperature suitability for Zika transmission in the Americas, we mapped the number of months for which *R*_0_(*T*) > 0 for the posterior median response, based on the temperature-dependent model derived here and previously [8]. This conservative threshold of *R*_0_(*T*) > 0 illustrates all pixels in which transmission is theoretically possible (given that the mosquito and pathogen are present), but not necessarily stable. We calculated *R*_0_(*T*) at 0.1°C increments and projected it onto the landscape for monthly mean current temperatures from WorldClim data at a 5 min resolution (approximately 10 km^2^ at the equator). This calculation gives a snapshot of the relative temperature suitability for transmission in each pixel each month, but does not account for the influence of short- or long-term variation in temperature. Climate data layers were extracted for the geographical area and defined using the Global Administrative Boundaries Databases [32]. All map calculations and manipulations were run in R using packages ‘raster’ [33], ‘maptools’ [34] and ‘Rgdal’ [35], following methods described in [36]. Resulting GeoTiffs were rendered in ArcGIS 10.3.1 [37], and mapped as figures. We display the difference between a previous model parametrized on the *Ae. aegypti*–DENV system [8] and our current predictions. This model was then validated using spatially explicit ZIKV case records from Columbia reported at the municipality level [38,39] (see Methods and Results in the electronic supplementary material).

## 3. Results

Our GLMM analysis found that our data and response variables (probability of infection, dissemination, infectiousness and dissemination efficiency) were best explained by temperature, dpi and their interaction. Further, the best model for all of our response variables was the full model (electronic supplementary material, tables S1 and S2) containing both linear and squared terms for temperature and dpi, as well as their interactions. This model captured the observed delayed ZIKV infection dynamics at the cool temperatures and the observed declines in ZIKV infection over time in the warmer temperature treatments owing to increased mosquito mortality. Our best model suggests that the effects of temperature and dpi combine to shape relative *R*_0_ (i.e. predicted risk of transmission for ZIKV), which differs from previous estimates generated from DENV-specific models.

### (a) The effect of temperature on Zika virus infection and infection dynamics

**Figure 1.**
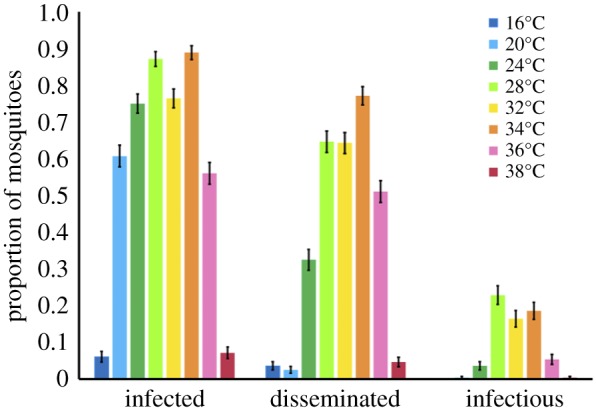
Temperature effect on the proportion of mosquitoes infected, with disseminated infections, and infectious. The effect of eight different constant temperatures (16°C, 20°C, 24°C, 28°C, 32°C, 34°C, 36°C, 38°C) on the proportion of mosquitoes infected (ZIKV positive bodies compared to total number of exposed), with disseminated infections (ZIKV positive heads compared to total number exposed), and infectious (ZIKV positive saliva compared to total number exposed).

We observed strong, unimodal effects of temperature on the number of mosquitoes infected, with disseminated infections, and that became infectious (figure 1; electronic supplementary material, table S1). While all three response variables dropped at both cool and warm temperatures, this decrease was more pronounced as the infection progressed (figure 1). The likelihood of becoming infected was the most permissive to temperature variation, with the number of infected mosquitoes minimized at 16°C (6%), maximized from 24°C–34°C (75%–89%), and again minimized at 38°C (7%). The likelihood of viral dissemination was more constrained, with the probability of mosquitoes disseminating infections minimized at 16–20°C (4%–3%), maximized at 28–34°C (65%–77%), and again minimized at 38°C (5%). Finally, the likelihood of mosquitoes becoming infectious was the most sensitive to temperature, with the probability of mosquitoes becoming infectious minimized from 16°C to 24°C (0%–4%), maximized between 28°C and 34°C (23%–19%), and again minimized from 36°C to 38°C (5%–0.4%).

**Figure 2.**
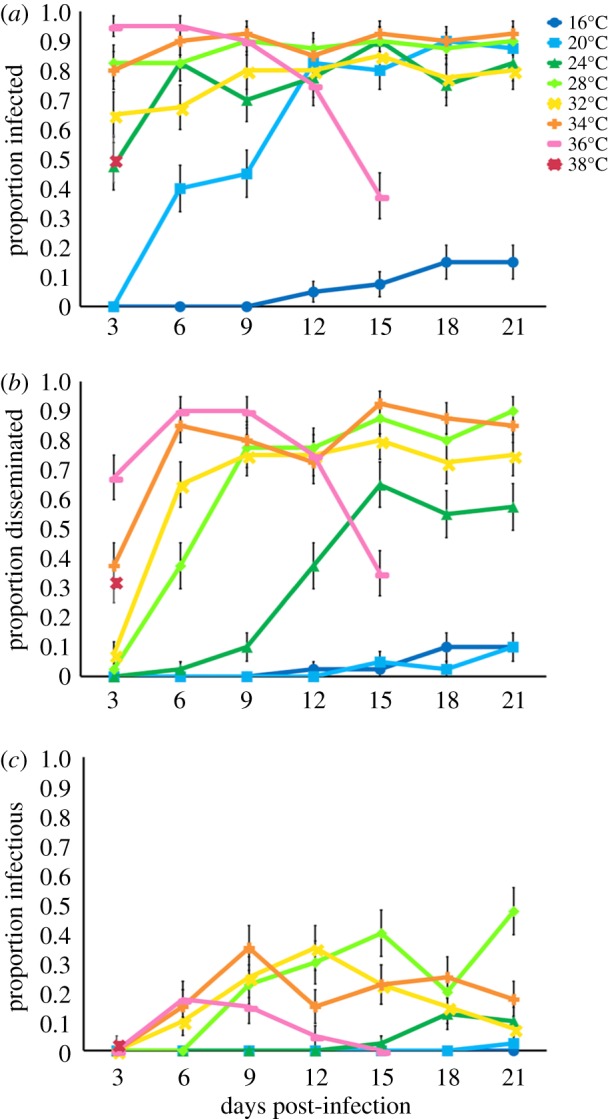
Days post-infection and the proportion of mosquitoes infected, with disseminated infections, and infectious. The relationship between days post-infection (3, 6, 9, 12, 15, 18, 21) and the proportion of mosquitoes infected (*a*, ZIKV positive bodies), with disseminated infections (*b*, ZIKV positive legs and heads), and infectious (*c*, ZIKV positive saliva) out of the total mosquitoes exposed to ZIKV at eight different constant temperatures (16°C, 20°C, 24°C, 28°C, 32°C, 34°C, 36°C, 38°C).

Temperature also affected the rate of ZIKV infection, dissemination, and detection in saliva (figure 2; electronic supplementary material, table S1). In general (with the exception of 36°C and 38°C), we observed an increase in the probability of mosquitoes with ZIKV in the bodies, legs and heads, and saliva with time (figure 2) suggesting that rate of ZIKV detection in these samples decreased with increasing temperature. However, at 36°C and 38°C, we see declines in these response variables with dpi owing to high mosquito mortality.

### (b) The effects of temperature on Zika virus dissemination efficiency

We observed effects of temperature, dpi and their interaction on the dissemination efficiency of ZIKV (the probability of becoming infectious after successful ZIKV infection–positive bodies; electronic supplementary material, table S2). ZIKV dissemination efficiency was maximized from 28°C to 34°C, suggesting that ZIKV infection process (e.g. escape from the midgut and salivary gland invasion) was most efficient at these temperatures (electronic supplementary material, figure S2). By contrast, dissemination efficiency was minimized at both cooler (16–20°C) and warmer temperatures (38°C). Cooler temperatures had a more dramatic effect on dissemination efficiency than warmer temperatures. Although 60% of exposed mosquitoes became successfully infected at 20°C, we had very low salivary gland invasion, with only one mosquito across both trials becoming infectious. By contrast, at warm temperatures infection and dissemination efficiencies were very robust (electronic supplementary material, figure S3), but the mortality associated with the warm temperatures resulted in low numbers of mosquitoes that were capable of being infectious. Finally, of those successfully infected, we observed successful salivary gland invasion to occur earlier in the infection process as temperatures warmed (electronic supplementary material, figure S2).

### (c) The effect of temperature on mosquito survival

We observed effects of temperature and an interaction between temperature and ZIKV exposure on the daily probability of mosquito survival (electronic supplementary material, figure S4 and table S3). Overall, the daily probability of mosquito survival was highest for mosquitoes housed at 24°C and 28°C relative to cooler (16–20°C) and warmer (32–38°C) temperatures. Mosquito survival was lowest at the warmest temperature of 38°C, with no mosquitoes surviving past 3 dpi. ZIKV-exposed mosquitoes experienced a higher daily probability of survival at 24°C and 28°C relative to unexposed, control mosquitoes with greater than 90% daily survival at the optimal temperatures.

### (d) The effect of temperature on Zika virus transmission risk

**Figure 3.**
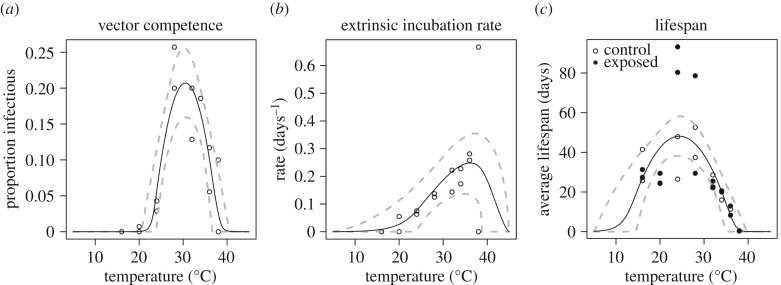
Effect of temperature and estimated vector competence, extrinsic incubation rate and mosquito lifespan. Trait thermal responses, fitted from laboratory experimental data. Vector competence (*a*), is the average proportion of virus-exposed mosquitoes with virus in their saliva, across temperatures and replicates. Extrinsic incubation rate (*b*) is the inverse of the time required to reach half of the average proportion infectious (days^−1^) for each temperature and replicate. Lifespan is the average lifespan of mosquitoes in each temperature and replicate (days), shown in filled (virus-exposed) and open (sham-inoculated) points. Solid lines represent posterior means; dashed lines represent 95% credible intervals.

**Figure 4.**
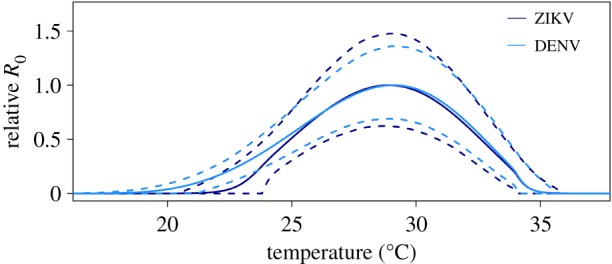
Effect of temperature on *R*_0_. Effect of temperature on relative *R*_0_ for DENV (light blue) and ZIKV (dark blue). Solid lines represent the mean and dashed lines are the 95% credible intervals.

**Figure 5.**
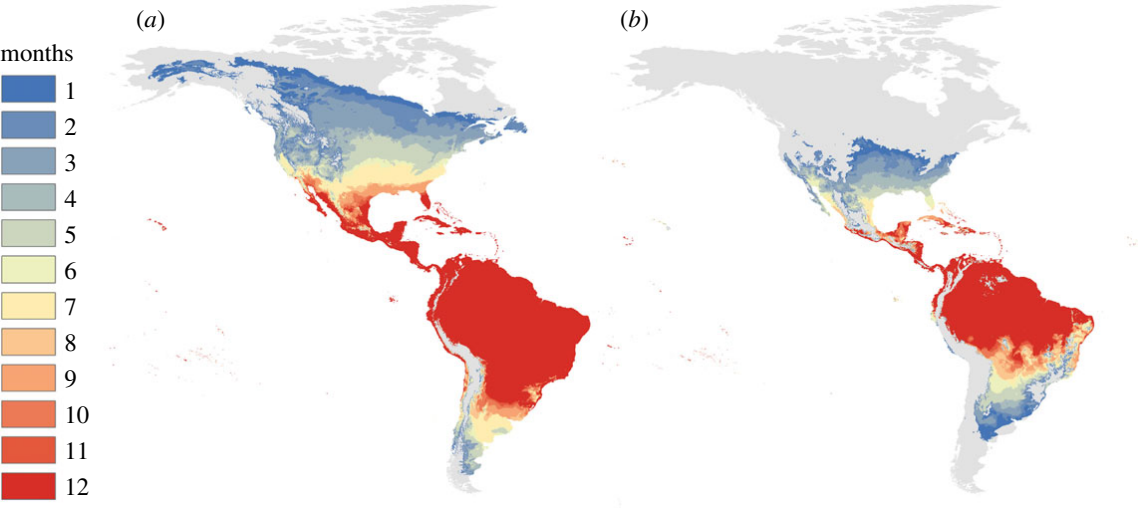
Months of transmission suitability in the Americas. The number of months of transmission suitability (*R*_0_ > 0) for DENV derived in Mordecai *et al.* [8] (*a*) and ZIKV derived in this study (*b*), for median (posterior 50th percentile) models.

Trait thermal responses for lifespan, vector competence and extrinsic incubation rate were all unimodal (figure 3; electronic supplementary material, table S4 and figure S5). Mosquito lifespan and vector competence thermal responses were symmetrical, peaking at 24.2°C (95% credible interval (CI): 21.9–25.9°C) and 30.6°C (95% CI: 29.6–31.4°C), respectively, while the extrinsic incubation rate thermal response was asymmetrical with a peak at 36.4°C (95% CI: 33.6–39.1°C). Applying these new trait thermal responses to the *R*_0_(*T*) model [8], we found that *R*_0_(*T*) peaked at 28.9°C (95% CI: 28.1–29.5°C), with lower and upper limits of 22.7°C (95% CI: 21.0–23.9°C) and 34.7°C (95% CI: 34.1–35.8°C), respectively (figure 4). The seasonal transmission of ZIKV was predicted to be more constricted in latitudinal range from this temperature–transmission relationship than what has been predicted previously [8], primarily because the predicted thermal minimum for ZIKV was 5°C warmer than for DENV (figure 4). This represents a 4.3 million km^2^ estimated change in endemic (12 month, year-round suitability) land area, and a 6.03 million km^2^ change in overall predicted range (1–12 months suitability) in the Americas (figure 5).

The spatial validation for Columbia showed that 71.5% (67 934) of all Zika cases fell within 1–12 months of predicted suitability, with 68% (64 286) ZIKV cases overlaid areas predicted to have 12 months of suitability. By contrast, our spatial validation predicted 28.5% (27 041) ZIKV cases to occur in areas predicted to be unsuitable for transmission (0 months of suitability, electronic supplementary material, figure S7). Upon visual inspection, large clusters of cases occurred in valleys where the *R*_0_ model predicted transmission suitability.

While there is some evidence that mosquito longevity varies for virus-exposed versus control mosquitoes, where unexposed mosquitoes had shorter lifespans at near-optimal temperatures (24°C and 28°C; figure 3; electronic supplementary material, figure S4), we did not include this difference in the *R*_0_ model for two reasons. First, with limited data to parametrize the low temperature range for survival, we are unable to characterize the differences in the lower end of the thermal response functions in detail. Second, the standard *R*_0_ model does not incorporate differences in survival for infected versus uninfected mosquitoes because it assumes that the pathogen is rare and that all mosquitoes are uninfected. For this reason, we fit a single thermal response function for lifespan to the full dataset and used it in the *R*_0_ model.

## 4. Discussion

The dynamics and distribution of vector-borne diseases depend on the interplay between the pathogen, the mosquito and the environment [40]. Temperature is a strong driver of vector-borne disease transmission, and characterizing the thermal range and optimum for transmission is essential for accurately predicting how arbovirus emergence and transmission will be affected by seasonality, geography, climate and land use change. Yet current models of recently emerging arboviruses like ZIKV are constrained by a lack of data on the thermal sensitivity of key pathogen traits (e.g. [6,7,9]). In this study, we experimentally estimated the relationship between temperature and measures of ZIKV vector competence, extrinsic incubation rate, and mosquito mortality. By incorporating these temperature-trait relationships into an existing mechanistic model, we demonstrate that, like malaria [15,41] and DENV virus [8], ZIKV transmission has a strong unimodal relationship with temperature.

As studies have demonstrated in other arbovirus systems, temperature significantly affects vector competence [16,42,43]. We show that temperature has a unimodal relationship with vector competence, with an estimated optimum at 30.6°C and an estimated thermal minimum and maximum of 22.9°C and 38.4°C, respectively (based on posterior median estimates for *T*_0_ and *T*_m_). ZIKV infectiousness was limited by different mechanisms at the thermal minimum and maximum. Cool temperatures limited midgut escape and dissemination, resulting in a lower proportion of the mosquito population that was infectious. This could be owing to temperature effects on mosquito physiology [44], immunity [12,45], and viral binding to specific receptors in the midgut, secondary tissues, and salivary glands [46]. Warmer temperatures, on the other hand, were very permissive for ZIKV infection, resulting in 95% and 100% infection among surviving mosquitoes at 36°C and 38°C, respectively (electronic supplementary material, figure S3). However, high mosquito mortality at these temperatures constrained the proportion of the mosquito population that became infectious (figure 2; electronic supplementary material, figure S3) A similar nonlinear effect of cool and warm temperatures on vector competence was observed with *Ae. albopictus* infected with DENV-2 [47]. By contrast, Adelman *et al*. [13] demonstrated that cooler temperatures in the larval stage resulted in increased susceptibility to CHIKV and yellow fever virus by impairing the RNAi pathway. However, mosquitoes in our study were exposed to different constant temperatures in the adult stage. Temperature variation experienced in both the larval and adult stage will probably be important in shaping mosquito and pathogen traits comprising arbovirus transmission.

We also observed an asymmetrical unimodal relationship between temperature and the extrinsic incubation rate of ZIKV, with the extrinsic incubation rate optimized at 36.4°C and minimized at 19.7°C and 42.5°C (based on posterior median estimates for *T*_0_ and *T*_m_). Consistent with previous studies (e.g. [43,47,48]), we show that the extrinsic incubation rate of ZIKV increased with warming temperatures, with no infectious mosquitoes observed at 16°C after 21 days post infection and the first infectious mosquito detected at day 3 post infection at 38°C. The extrinsic incubation rate was ultimately constrained at the warmer temperatures owing to high mosquito mortality. This is not surprising as metabolic reaction rates tend to increase exponentially to an optimal temperature, then decline rapidly owing to protein degradation and other processes [21,49].

The optimal temperature for mosquito fitness and viral dissemination need not be equivalent, and the impacts of temperature on mosquito mortality relative to the extrinsic incubation rate of arboviruses strongly affect the total proportion of the mosquito population that is alive and infectious [50,51]. In our study, mosquito lifespan was optimized at 24.2°C and minimized at 11.7°C and 37.2°C, respectively (based on posterior median estimates for *T*_0_ and *T*_m_). The nonlinear relationship between metrics of mosquito mortality or lifespan and temperature has also been demonstrated for *Ae. aegypti* [8], *Aedes albopictus* [8,14] and various *Anopheles* spp. [15,52]. Despite the fact that the extrinsic incubation rate was optimized at a warm temperature (36.4°C), the optimal temperature for overall ZIKV transmission (*R*_0_) was predicted to be cooler (28.9°C) because mosquitoes experience a shortened lifespan above 32°C. By contrast, even though mosquitoes are predicted to have relatively longer lifespans at cooler temperatures, the time required for mosquitoes to become infectious (greater than 21 days at 16°C and 18 days at 20°C) may be longer than most mosquitoes experience in the field. As a result, large vector populations may not be sufficient for transmitting the virus if viral replication is inhibited or if the lifespan of the mosquito is shorter than the extrinsic incubation period [53]. One surprising result was that mosquitoes exposed to ZIKV were predicted to live significantly longer than unexposed mosquitoes at temperatures already optimal for mosquito survival (37 versus 87 days at 24°C; 45 versus 54 days at 28°C). A similar phenomenon has been noted in the *Ae. aegypti*–DENV-2 system [54]. Additionally, the temperature that optimizes mosquito lifespan might also vary between ZIKV exposed mosquitoes (24°C) and their uninfected counterparts (28°C). However, more data characterizing mosquito survival of uninfected and ZIKV exposed mosquitoes at the cool range of transmission are needed to better understand the consequences of survival differences between ZIKV infected and uninfected mosquitoes. If temperature consistently has different effects on the mortality rates of uninfected and infected mosquitoes in other arbovirus systems, current modelling efforts may be underestimating virus transmission potential under certain environmental scenarios and estimating mosquito mortality in the field for mosquitoes of different infection statuses are important areas for future research.

After incorporating the relationships between temperature and vector competence, the EIR, and mosquito lifespan into a mechanistic model, we demonstrated that ZIKV transmission is optimized at a mean temperature of approximately 29°C, and has a thermal range of 22.7°C to 34.7°C. Because this relationship is nonlinear and unimodal, we can expect as temperatures move toward the thermal optimum owing to future climate change or increasing urbanization [55], environmental suitability for ZIKV transmission should increase, potentially resulting in expansion of ZIKV further north and into longer seasons. There is evidence that this is already occurring with warming at high elevations in the Ethiopian and Columbian highlands leading to increased incidence of malaria [11]. By contrast, in areas that are already permissive and near the thermal optimum for ZIKV transmission, future warming and urbanization may lead to decreases in overall environmental suitability [17]. Accurately estimating the optimal temperature for transmission is thus paramount for predicting where climate warming will expand, contract, or shift transmission potential.

By using a mechanistic model originally parametrized for DENV (data from serotypes 1 and 2), we also explored a common assumption made by multiple models that DENV transmission has a similar relationship with temperature as ZIKV [6–9,20]. While the temperature optimum and maximum for *R*_0_ changed very little from our previous DENV *R*_0_ model, the temperature minimum for transmission increased by nearly five degrees in the ZIKV-specific model (figure 4). This is mainly owing to a higher thermal minimum for both vector competence and the extrinsic incubation rate for ZIKV as compared to DENV (electronic supplementary material, figure S6). Differences in the thermal niche of ZIKV relative to DENV or our field derived *Ae. aegypti* relative to those populations synthesized in Mordecai *et al*. [8] could explain this difference. There is evidence that the effects of environmental variation on disease transmission are often modified by the genetic background of the mosquito and infecting pathogen [42,56,57]. Thus, more work is required to validate the generalizability of these models.

Our mapped seasonal ranges underscore the impact of a more refined empirical derivation of a pathogen-specific temperature dependent *R*_0_, contrasted with the *Ae. aegypti* DENV prediction of previous studies [6–8]. The higher predicted thermal minimum for ZIKV resulted in a contraction in the areas of the Americas where year-round, endemic transmission suitability (12 months only) are predicted to occur. This area corresponds to a change of approximately 4.3 million km^2^ in land area (figure 5). Additionally, this higher thermal minimum contributes to a reduction in the overall estimated suitability for ZIKV transmission (all 1–12 months of transmission) resulting in an estimated difference of 6.03 million km^2^. In particular, in the Florida peninsula where the primary focus of ZIKV cases within the US occurred, our updated model (the median model—50th percentile posterior) now predicts only around six months of temperature suitability during the year (figure 5) versus almost year-round as predicted by a previous temperature-dependent *R*_0_ model parametrized on the *Ae. aegypti*–DENV system [8]. This contrast in seasonal suitability where ZIKV established in the USA is striking, and emphasizes the value of increasing empirical data and re-examining these types of models, as the capacity to do so becomes possible, in the face of an emerging epidemic. This result also largely concurs with a previous study that generated an *R*_0_ ZIKV map of the Americas using a spatially explicit individual based susceptible, exposed, infected–susceptible, exposed, infected, recovered compartmental model. This model incorporates unimodal temperature-trait relationships for mosquito lifespan, probability of transmission parametrized from the DENV-*Ae. aegypti* system, and mosquito abundance with pre-existing data layers for *Ae. aegypti* and *Ae. albopictus* distributions, spatially explicit human population and economic data, and ZIKV case data [20]. While our models generally agree in the geographical extent to which ZIKV transmission is predicted to occur in the Americas, Zhang *et al*. show more heterogeneity in *R*_0_ across space than our model would predict, which simply describes the temperature boundaries for potential ZIKV transmission on the landscape. However, our model, which uses a broader life-history explicit parametrization including ZIKV-specific thermal responses for relevant transmission, provides important validation of the predictions in Zhang *et al*. [20].

Our spatial validation of this model revealed fairly robust predictive qualities (electronic supplementary material, figure S7), despite limitations of spatial resolution and scale at which ZIKV cases are reported. For example, aggregating at the municipality scale created a much larger minimum areal unit than the model pixels. Further, this region is subject to high variation in local conditions owing to the Andes climate, which are probably not accurately captured by global temperature model outputs. Thus, we might expect the *R*_0_ model to over or under predict fine-scale variation in local microclimate [54]. Regardless, this highlights a need for more spatially detailed health datasets to be available to this type of model validation exercise, as well as regionalized modelling efforts in climate-health initiatives.

Finally, although we estimated the effects of mean constant temperatures on ZIKV transmission, mosquitoes and their pathogens live in a variable world where temperatures fluctuate daily and seasonally. Temperature-trait relationships have been shown to differ in fluctuating environments relative to constant temperature environments (e.g. [17,58,59]). While characterizing trait responses to mean constant temperatures and incorporating these relationships into models of disease transmission is tractable, more effort is needed in validating computational approaches to infer transmission in a fluctuating environment (i.e. rate summation [8,60]).

Accurately predicting arbovirus transmission will be influenced by variation in other sources of abiotic (e.g. relative humidity, rainfall), biotic (e.g. availability and quality of oviposition and resting habitats), and socioeconomic factors that influence human exposure to biting mosquitoes [20]. However, this is a fundamental first step for empirically defining and validating current models on the environmental suitability for ZIKV transmission, in which temperature will be a strong driver. *R*_0_ models have been used as a tool to guide vector-borne disease interventions, and represent a comprehensive metric of pathogen fitness. We anticipate, as with other vector-borne diseases, that environmental suitability for ZIKV transmission could expand northwards with future warming, but will be more constrained than DENV at low temperatures. We also predict areas that are already at or near the thermal optimum of 29°C to experience a decrease in environmental suitability for ZIKV transmission [15,17]. Further, land use change that modifies the microclimates mosquitoes experience and human density and exposure could have immediate impacts on ZIKV transmission, which might explain the explosive spread of ZIKV in urban centres throughout the Americas.

## Supplementary Material

Methods and Results SI

## Supplementary Material

Figures SI

## Data accessibility

Data and code are available from the Dryad Digital Repository: http://dx.doi.org/10.5061/dryad.7hj6q4c [61].
