# Supplementary material for: Temperature drives Zika virus transmission: evidence from empirical and mathematical models
Source: Proc Biol Sci. 2018 Aug 15;285(1884):20180795. doi: 10.1098/rspb.2018.0795 (PMC6111177; doi:10.1098/rspb.2018.0795)
Supplement: Methods and Results SI [file rspb20180795supp1.docx]

**Supplementary Information**

**Methods**

**Virus culture**

We used the ZIKV isolate MEX1-44 obtained from the University of Texas Medical Branch (UTMB) Arbovirus Reference Collection. The virus was isolated in January 2016 from a field-caught *Ae. aegypti* mosquito from Tapachula, Chiapas, Mexico. For all mosquito infections, we used pass ten stock virus that was passaged four times at the UTMB and an additional six times at the University of Georgia. Four days after inoculation in Vero cells, we harvested the virus, centrifuged it at 2,500xg for 5 min, and stored it at -80°C. The virus tested negative for *Mycoplasma* contamination using MycoSensor PCR Assay Kit (Agilent) and was titrated using standard plaque assays on Vero cells [1]. Briefly, we infected the cells with six 10-fold serial dilutions for 1-2 hours. After incubation, we removed the inoculum and replaced it with 1.5% agarose DMEM (UltraPure LMP Agarose, Fisher Scientific). The cells were kept at 37°C, 5% CO_2_ for four days when they were fixed with 4% formalin and stained with crystal violet. The titers were expressed in plaque forming units per milliliter (PFU/mL).

**Mosquito rearing**

Outbred *Ae. aegypti* mosquito colonies were generated from ovitrap collections in Tapachula, Chiapas, Mexico, spring 2016. Mosquito eggs were hatched in ddH_2_O under reduced pressure in a vacuum desiccator and dispersed larvae in rearing trays. Each tray contained 200 larvae in 1L ddH_2_O and 4 fish food pellets (Hikari Cichlid Gold Large Pellets). Adult mosquitoes were kept in rearing cages and provided with 10% sucrose *ad libitum*. We maintained the colonies on whole human blood (Interstate Blood Bank) and collected eggs on paper towels. Larvae and adults were maintained under standard, controlled insectary conditions at 27°C ± 0.5°C, 80% ± 10% relative humidity, and a 12:12 light: dark diurnal cycle in a dedicated environmental walk-in room (Percival Scientific).

**Temperature Dependent Ro Model Methods**

Experimental data on lifespan, vector competence, and extrinsic incubation rates were used across temperatures to estimate trait thermal response functions for calculating *R_0_*(*T*). Because we destructively sampled mosquitoes to assess infection status and did not follow all mosquito cohorts to the end of their lifespan, we used Gompertz survival curves to estimate average lifespan. First, Kaplan-Meier daily probabilities of survival for each experimental replicate, infection status, and temperature were estimated. Then, we used the ‘nls’ function in R [2] to fit a Gompertz function to the daily survival probabilities for each infection status by trial and temperature combination. To estimate the average female lifespan of exposed and control mosquitoes for each temperature treatment and experimental replicate, we calculated the area under the curve by integrating the associated Gompertz function. Vector competence for each temperature was estimated from the average proportion of mosquitoes observed to become infectious at each temperature. For estimating the ZIKV extrinsic incubation rate (*EIR*) at each temperature, we calculated the time required for half of the average proportion of the population to become infectious (and defined this as the average extrinsic incubation period, *EIP*), then inverted this time interval to estimate a daily rate of ZIKV development for each temperature (*1/EIP*).

Using these data, we fit thermal response functions for lifespan, EIR, and vector competence as either symmetric (Quadratic: *-c*(*T-T_0_* )(*T-T_m_* )) or asymmetric (Briere: *cT*(*T-T_0_*) (*T_m_-T*)*^*(*1⁄2*)) functions, where *T* is experimental temperature, *T_0_* is the minimum temperature, *T_m_* is the maximum temperature, *c* is a rate constant, and both functions are truncated at zero for negative values [3, 4]. As in previous work [14], we fit the thermal response functions using Bayesian inference with uninformative priors, which are restricted to biologically reasonable ranges: *T_0_* ~ Uniform (0, 24), *T_m_* ~ Uniform (25, 45), *c* ~ Gamma (1, 10) for Briere and *c* ~ Gamma (1, 1) for Quadratic [4]. In the model, we assume that the sampling process is a normal distribution centered on the thermal response function calculated at the experimental temperature, with precision *τ* (where *τ=1/σ*) assigned the prior: Gamma ~ (0.0001, 0.0001). We fit the models using JAGS [5] and R [2] and the R package ‘rjags’ [6], by running five Markov Chain Monte Carlo simulations for a 5,000-step burn-in followed by 5,000 additional steps, then thinning the posterior samples by saving every fifth sample [3, 4].

The three new thermal response functions (*lf, EIR,* and *bc*) were combined with the remaining previously-fitted thermal response functions [4] to calculate *R_0_*(*T*) for ZIKV. To do so, we propagated the posterior distribution of each parameter thermal response through the *R_0_*(*T*) function to calculate a posterior distribution on *R_0_*(*T*).

**Spatial Validation of Temperature Dependent *R_0_* Maps**

We performed a spatial join to mapped administrative boundaries for municipalities in Colombia, and summed reported Zika cases at the level of municipality for the entirety of the dataset (total cases=94,975). We overlaid this municipal level data onto the model predictions for months suitable for ZIKV transmission as a function of temperature. In absence of finer scale data than municipality level reporting, we simply extracted the model pixel value at the geographic centroid of each municipality represented, and report the number and proportion of ‘hits’ and ‘misses’ of cases.

**References**

1. Willard K.A., Demakovsky L., Tesla B., Goodfellow F.T., Stice S.L., Murdock C.C., Brindley M.A. 2017 Zika virus exhibits lineage-specific phenotypes in cell culture, in *Aedes aegypti* mosquitoes, and in an embryo model. *Viruses* **9**(12). (doi:10.3390/v9120383).

2. R Core Team. 2017 R: A language and environment for statistical computing. (Vienna, Austria, R Foundation for Statistical Computing.

3. Johnson L.R., Ben-Horin T., Lafferty K.D., McNally A., Mordecai E., Paaijmans K.P., Pawar S., Ryan S.J. 2015 Understanding uncertainty in temperature effects on vector-borne disease: a Bayesian approach. *Ecology* **96**(1), 203-213.

4. Mordecai E., Cohen J., Evans M.V., Gudapati P., Johnson L.R., Lippi C.A., Miazgowicz K., Murdock C.C., Rohr J.R., Ryan S.J., et al. 2017 Detecting the impact of temperature on transmission of Zika, dengue, and chikungunya using mechanistic models. *PLoS Negl Trop Dis* **11**(4), e0005568. (doi:<https://doi.org/10.1371/journal.pntd.0005568>).

5. Plummer M. 2003 JAGS: A program for analysis of Bayesian graphical models using Gibbs sampling. In *Proceedings of the 3rd International Workshop on Distributed Statistical Computing* (Vienna, Austria.

6. Plummer M. 2016 rjags: Bayesian Graphical Models using MCMC. R package version 4-6. (

**Results**

**Table S1.** Results from candidate generalized linear mixed effects models (binomial distribution, log link function, random effect of mosquito cohort) examining the fixed effects of temperature, day, and the interaction on the probability of mosquitoes becoming infected, disseminating infection, and becoming infectious after being exposed to ZIKV. Bolded model outputs represent the most parsimonious model as determined by Akaike Information criterion scores corrected for small sample sizes (AICc).

| **Fixed Effects** | **df** | **AICc** | ***Intercept*** | ***T*** | ***dpi*** | ***T: dpi*** | ***T^2^*** | ***D^2^*** | ***T^2^: D^2^*** |
| --- | --- | --- | --- | --- | --- | --- | --- | --- | --- |
| Infected ~ *T* + *dpi* | 4 | 2418.30 | 0.228 | 1.268 | 0.026 |  |  |  |  |
| Infected ~ *T + T^2^ + dpi* | 5 | 2132.60 | 2.184 | -0.228 | 0.031 |  | -1.995 |  |  |
| Infected ~ *T + dpi + dpi^2^* | 5 | 2412.40 | 0.391 | 1.273 | 0.026 |  |  | -0.162 |  |
| Infected ~ *T + T^2^ + dpi + dpi^2^* | 6 | 2125.6 | 2.381 | -0.229 | 0.030 |  | -2.004 | -0.187 |  |
| Infected ~ *T* + *dpi* *+ T: dpi* | 5 | 2238.60 | 0.176 | 1.406 | 0.000 | -0.721 |  |  |  |
| Infected ~ *T + dpi + T: dpi + T^2^* | 6 | 1870.80 | 2.612 | -0.407 | 0.244 | -1.091 | -2.552 |  |  |
| Infected ~ *T + dpi + T:dpi + dpi^2^* | 6 | 2225.50 | 0.408 | 1.432 | -0.006 | -0.756 |  | -0.242 |  |
| **Infected ~ *T + dpi + T: dpi + T^2^ + dpi^2^ + T^2^: dpi^2^*** | **8** | **1861.60** | **2.804** | **-0.373** | **0.232** | **-1.115** | **-2.494** | **-0.226** | **-0.027** |
| Disseminated ~ *T* + *dpi* | 4 | 2330.70 | -0.791 | 1.724 | 0.332 |  |  |  |  |
| Disseminated ~ *T + T^2^ + dpi* | 5 | 2133.70 | 0.813 | 1.018 | 0.354 |  | -2.087 |  |  |
| Disseminated ~ *T + dpi + dpi^2^* | 5 | 2288.30 | -0.407 | 1.764 | 0.358 |  |  | -0.406 |  |
| Disseminated ~ *T + T^2^ + dpi + dpi^2^* | 6 | 2090.00 | 1.244 | 1.040 | 0.370 |  | -2.122 | -0.424 |  |
| Disseminated ~ *T* + *dpi* *+ T: dpi* | 5 | 2217.00 | -1.008 | 2.079 | 0.562 | -0.717 |  |  |  |
| Disseminated ~ *T + dpi + T: dpi + T^2^* | 6 | 1895.40 | 0.969 | 1.662 | 1.055 | -1.473 | -3.027 |  |  |
| Disseminated ~ *T + dpi + T:dpi + dpi^2^* | 6 | 2134.90 | -0.527 | 2.292 | 0.726 | -0.976 |  | -0.618 |  |
| **Disseminated ~ *T + dpi + T: dpi + T^2^ + dpi^2^ + T^2^: dpi^2^*** | **8** | **1782.70** | **2.021** | **1.922** | **1.244** | **-1.749** | **-3.837** | **-1.103** | **0.618** |
| Infectious ~ *T* + *dpi* | 4 | 1125.70 | -3.046 | 1.356 | 0.387 |  |  |  |  |
| Infectious ~ *T + T^2^ + dpi* | 5 | 1048.30 | -1.331 | 1.357 | 0.392 |  | -2.949 |  |  |
| Infectious ~ *T + dpi + dpi^2^* | 5 | 1101.80 | -2.626 | 1.371 | 0.530 |  |  | -0.527 |  |
| Infectious ~ *T + T^2^ + dpi + dpi^2^* | 6 | 1024.30 | -0.890 | 1.367 | 0.534 |  | -2.975 | -0.531 |  |
| Infectious ~ *T* + *dpi* *+ T: dpi* | 5 | 1111.90 | -3.242 | 1.679 | 0.592 | -0.542 |  |  |  |
| Infectious ~ *T + dpi + T: dpi + T^2^* | 6 | 1013.10 | -1.514 | 2.126 | 0.883 | -1.205 | -3.550 |  |  |
| Infectious ~ *T + dpi + T:dpi + dpi^2^* | 6 | 1079.00 | -2.859 | 1.877 | 0.900 | -0.848 |  | -0.648 |  |
| **Infectious ~ *T + dpi + T: dpi + T^2^ + dpi^2^ + T^2^: dpi^2^*** | **8** | **971.80** | **-0.953** | **2.667** | **1.461** | **-1.950** | **-4.259** | **-0.946** | **0.425** |

**Table S2.** Results from candidate generalized linear mixed effects models (binomial distribution, log link function, random effect of mosquito cohort) examining the fixed effects of temperature, day, and the interaction on the probability of mosquitoes becoming infectious after being successfully infected with ZIKV (positive body infection). Bolded model outputs represent the most parsimonious model as determined by Akaike Information criterion scores corrected for small sample sizes (AICc).

| **Fixed Effects** | **df** | **AICc** | ***Intercept*** | ***T*** | ***dpi*** | ***T: dpi*** | ***T^2^*** | ***D^2^*** | ***T^2^: D^2^*** |
| --- | --- | --- | --- | --- | --- | --- | --- | --- | --- |
| Infectious ~ *T* + *dpi* | 4 | 987.40 | -2.120 | 0.848 | 0.472 |  |  |  |  |
| Infectious ~ *T + T^2^ + dpi* | 5 | 945.70 | -1.149 | 0.713 | 0.448 |  | -1.193 |  |  |
| Infectious ~ *T + dpi + dpi^2^* | 5 | 966.50 | -1.719 | 0.868 | 0.598 |  |  | -0.485 |  |
| Infectious ~ *T + T^2^ + dpi + dpi^2^* | 6 | 926.00 | -0.742 | 0.716 | 0.571 |  | -1.210 | -0.478 |  |
| Infectious ~ *T* + *dpi* *+ T: dpi* | 5 | 984.80 | -2.310 | 1.034 | 0.567 | -0.277 |  |  |  |
| Infectious ~ *T + dpi + T: dpi + T^2^* | 6 | 927.70 | -1.180 | 1.074 | 0.727 | -0.707 | -1.630 |  |  |
| Infectious ~ *T + dpi + T:dpi + dpi^2^* | 6 | 952.80 | -1.988 | 1.290 | 0.891 | -0.642 |  | -0.648 |  |
| Infectious **~ *T + dpi + T: dpi + T^2^ + dpi^2^ + T^2^: dpi^2^*** | **8** | **891.20** | **-0.594** | **1.422** | **1.192** | **-1.224** | **-2.076** | **-0.867** | **0.250** |

**Table S3.** Results from Cox mixed-effects model examining the effects of temperature (16°C, 20°C, 24°C, 28°C, 32°C, 34°C, 36°C, 38°C), infection status (exposed or not exposed) and the interaction on the daily probability of mosquito survival.

| effect | Chi-Square | d.f. | *p*-value |
| --- | --- | --- | --- |
| temperature | 1138.226 | 7 | **<0.0001** |
| infection | 0.227 | 1 | 0.6338 |
| temperature x infection | 25.871 | 7 | **0.0005** |

**Table S4** Data used on the *Ae. aegypti* Zika virus *R_0_* model. Each trait parameter symbol, definition, data sources, and thermal response function (Quad = quadratic) are shown on the left. Mean and 95% credible interval (95% HPD interval) for the critical thermal minimum (*T_0_*), maximum, (*T_m_*), and a rate constant (*c*) are given for each trait in the three right sections.

^1^Mordecai E., Cohen J., Evans M.V., Gudapati P., Johnson L.R., Lippi C.A., Miazgowicz K., Murdock C.C., Rohr J.R., Ryan S.J., et al. 2017 Detecting the impact of temperature on transmission of Zika, dengue, and chikungunya using mechanistic models. *PLoS Negl Trop Dis* **11**(4), e0005568. (doi:<https://doi.org/10.1371/journal.pntd.0005568>).

^2^Estimated from data generated in the current study
