# Supplementary material for: Temperature drives Zika virus transmission: evidence from empirical and mathematical models
Source: Proc Biol Sci. 2018 Aug 15;285(1884):20180795. doi: 10.1098/rspb.2018.0795 (PMC6111177; doi:10.1098/rspb.2018.0795)
Supplement: Figures SI [file rspb20180795supp2.docx]

**Figure S1**


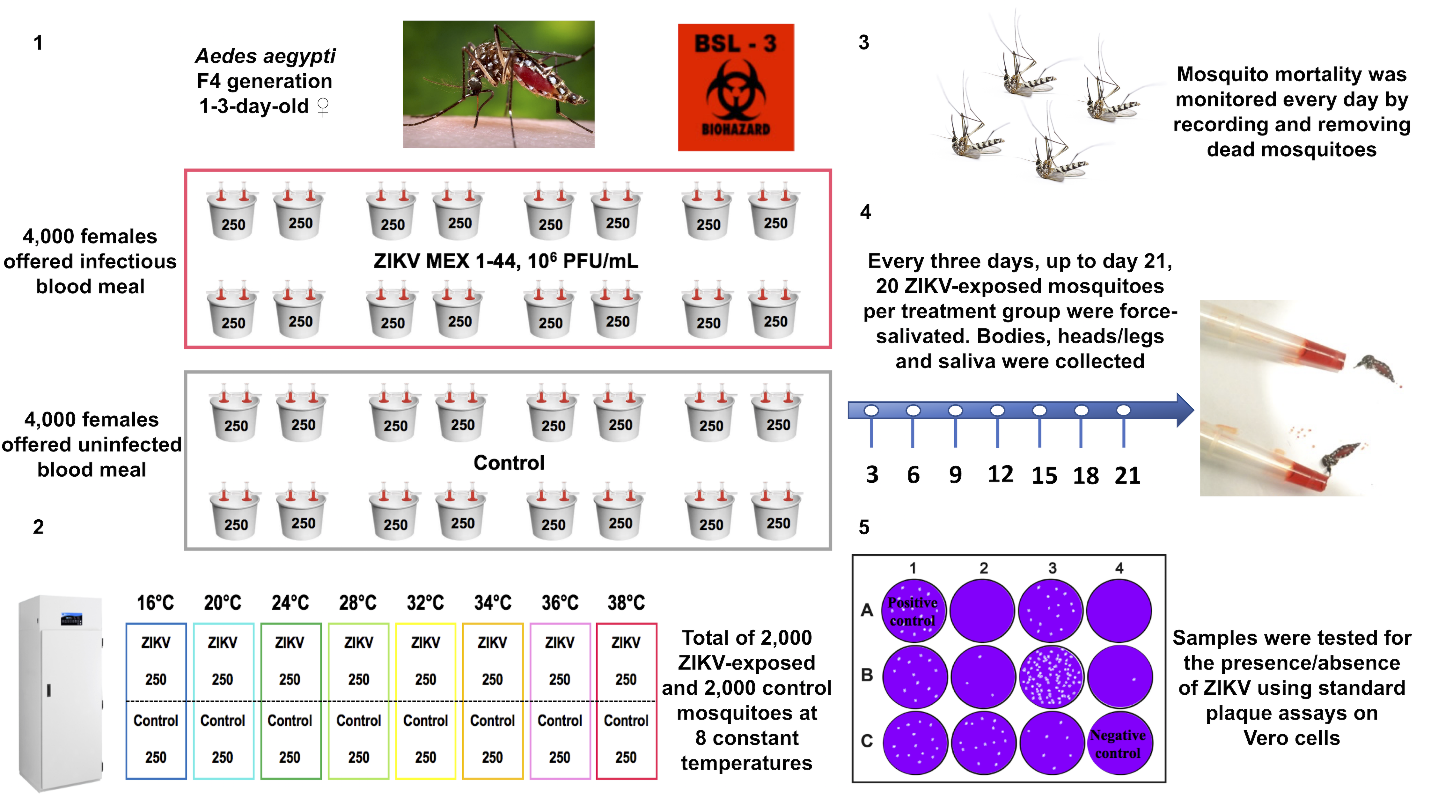


In each biological replicate, eight thousand female *Aedes aegypti* mosquitoes were offered either an infectious blood meal containing ZIKV at the final concentration of 10^6^ PFU/mL or an uninfected, control blood meal (1). Two thousand ZIKV-exposed and two thousand control engorged mosquitoes were randomly distributed into mesh-covered paper cups (250 per cup) and put at one of eight temperature treatments (16°C, 20°C, 24°C, 28°C, 32°C, 34°C, 36°C, and 38°C; 2). Across all treatment groups, dead mosquitoes were removed and counted daily to monitor mortality (3). Every three days, up to day twenty-one, twenty ZIKV exposed mosquitoes per treatment group were force-salivated. After salivation, mosquito saliva, heads, legs, and bodies were collected into separate tubes (4). Each tissue was tested for the presence/absence of the ZIKV using plaque assays on Vero cells (5). Two full biological replicates were performed.

**Figure S2**

Temperature effect on the dissemination efficiency (A) The effect of eight different constant temperatures (16°C, 20°C, 24°C, 28°C, 32°C, 34°C, 36°C, 38°C) and (B) days post-infection (3, 6, 9, 12, 15, 18, 21) on the dissemination efficiency (proportion of mosquitoes with ZIKV positive saliva relative to those with positive bodies).

**Figure S3**


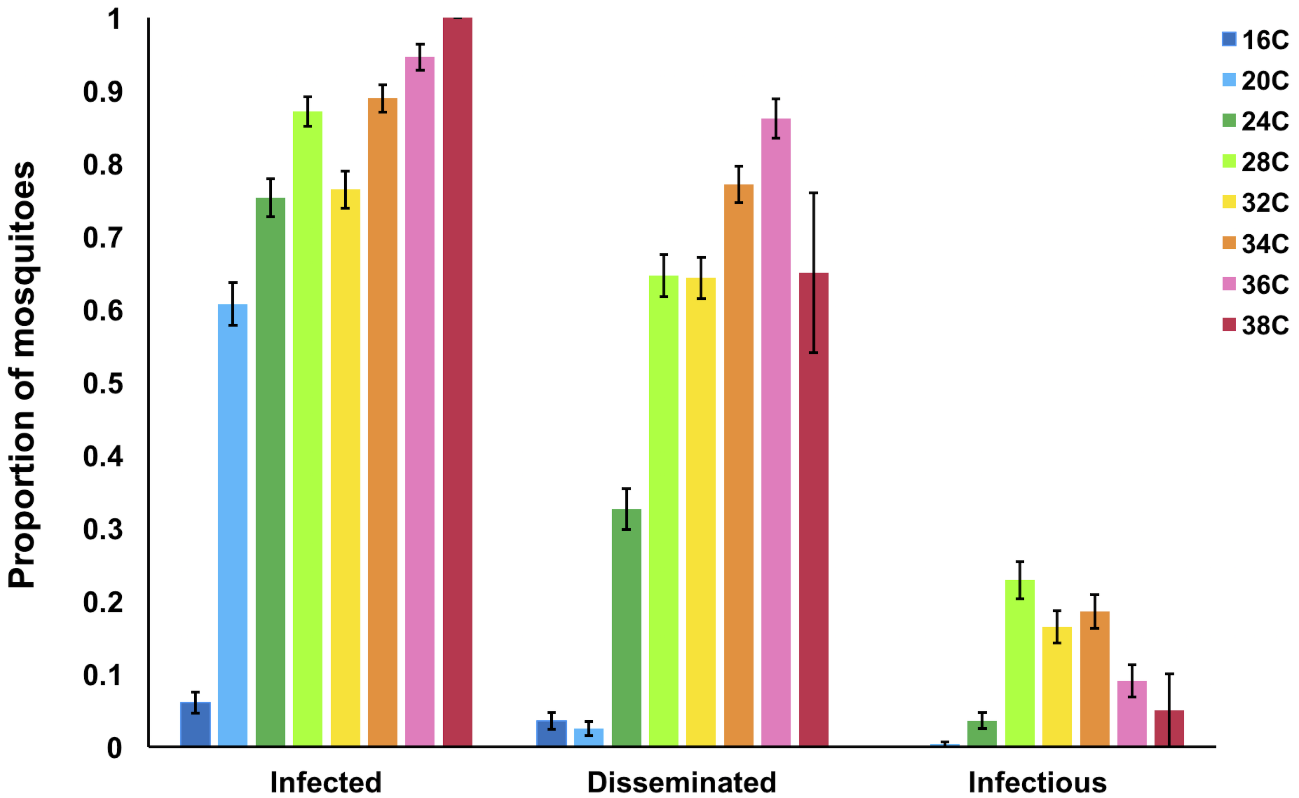


The effect of eight different constant temperatures (16°C, 20°C, 24°C, 28°C, 32°C, 34°C, 36°C, 38°C) on the proportion of mosquitoes infected (proportion of ZIKV positive bodies out of total number of processed mosquitoes), with disseminated infections (proportion of ZIKV positive heads out of total number of processed mosquitoes), and infectious (proportion of ZIKV positive saliva out of total number of processed mosquitoes).

**Figure S4**

**
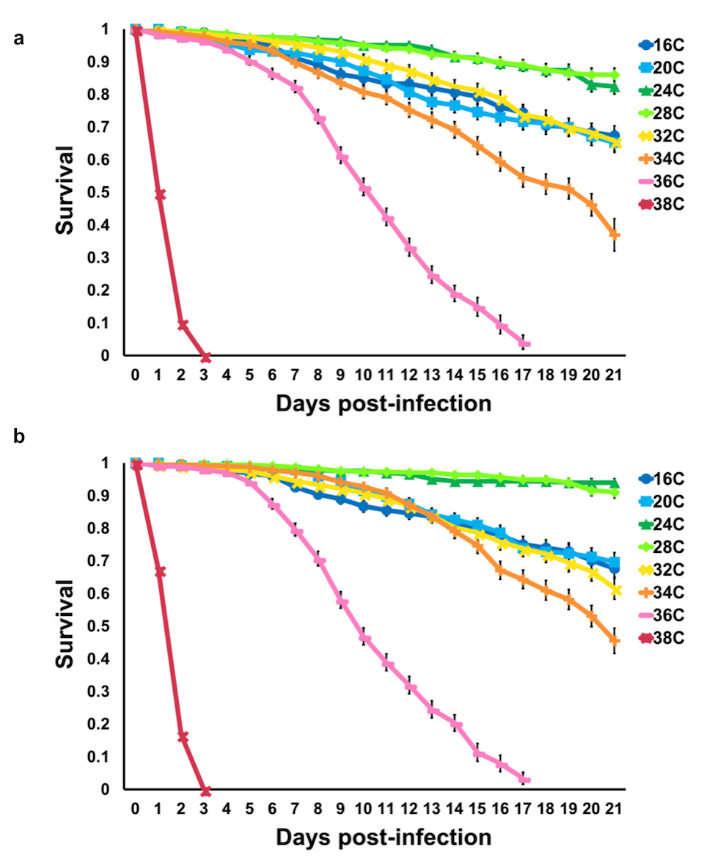
**

Kaplan-Meier estimates of daily probability of mosquito survival for unexposed (A) and ZIKV exposed (B) field-derived *Ae. aegypti* mosquitoes across eight different constant temperatures (16°C, 20°C, 24°C, 28°C, 32°C, 34°C, 36°C, 38°C).

**Figure S5**

**
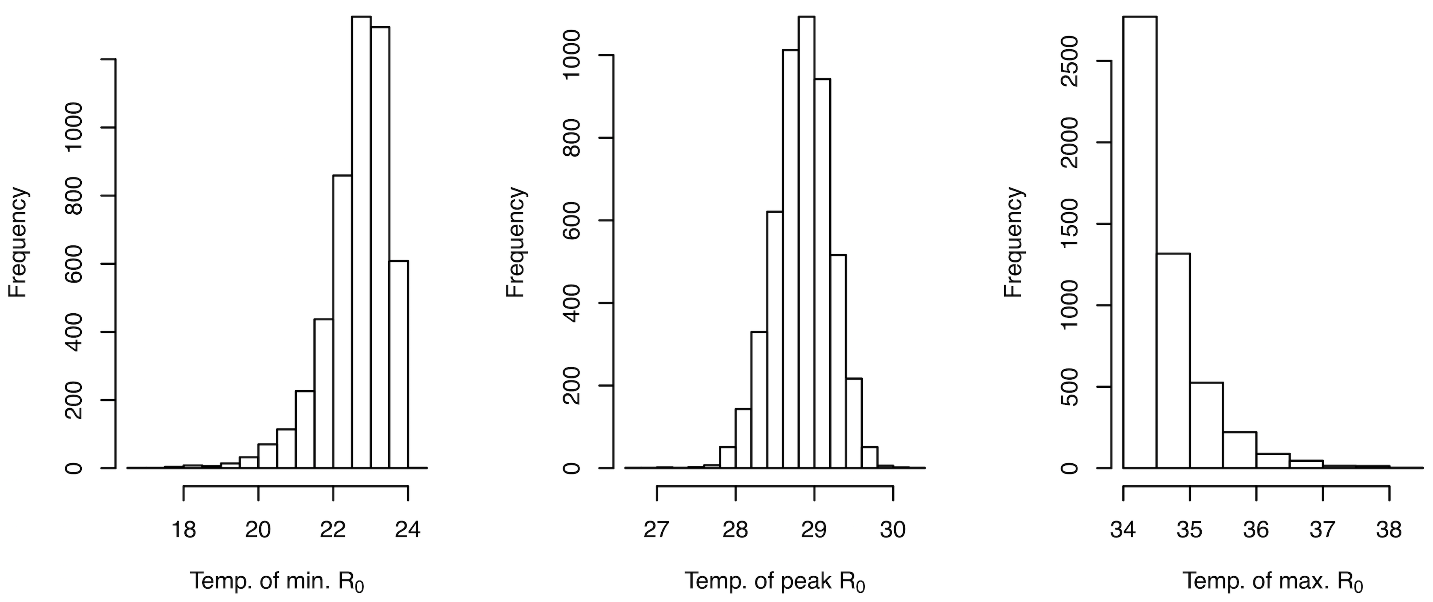
**

Histograms showing the posterior distribution of the temperature minimum (left), optimum (center), and maximum (right) for *R_0_*.

**Figure S6**

**
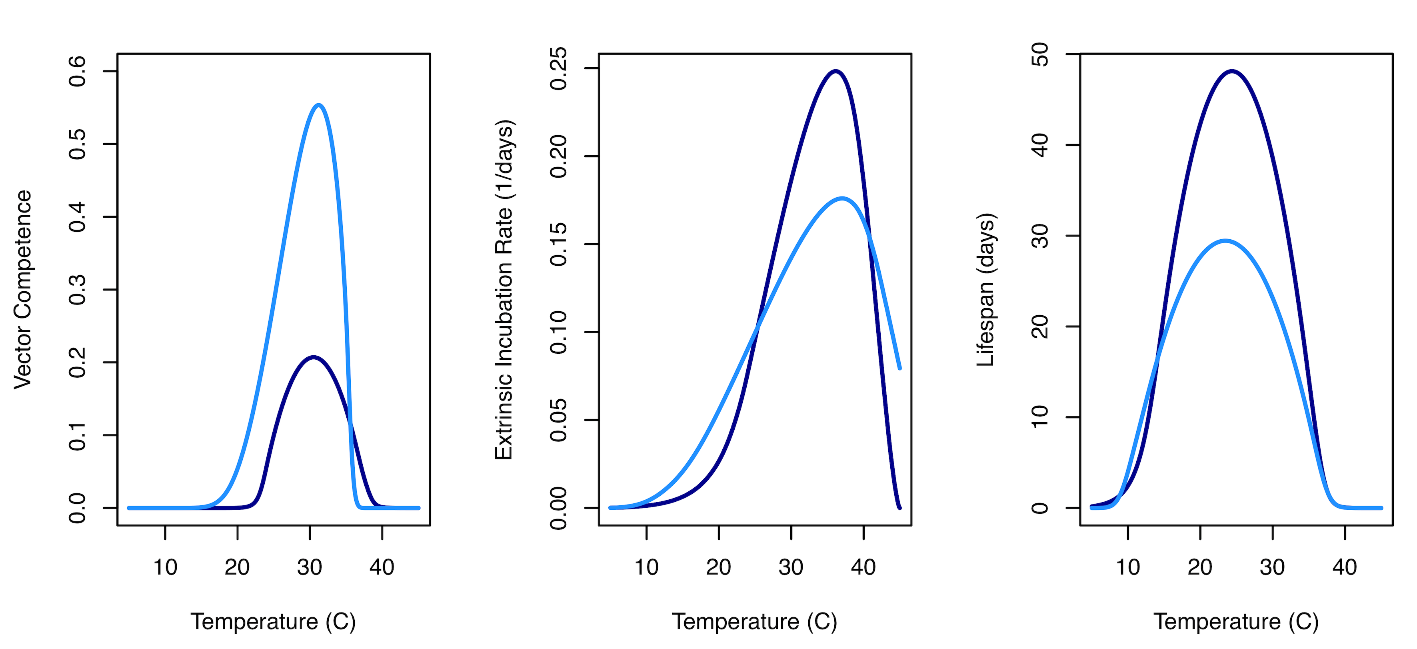
**

Trait thermal response means for vector competence (left), extrinsic incubation rate (center), and lifespan (right) for the new experimental data presented here (Zika virus; dark blue) and the previously published data (dengue virus, light blue) [1].

**Figure S7**

**
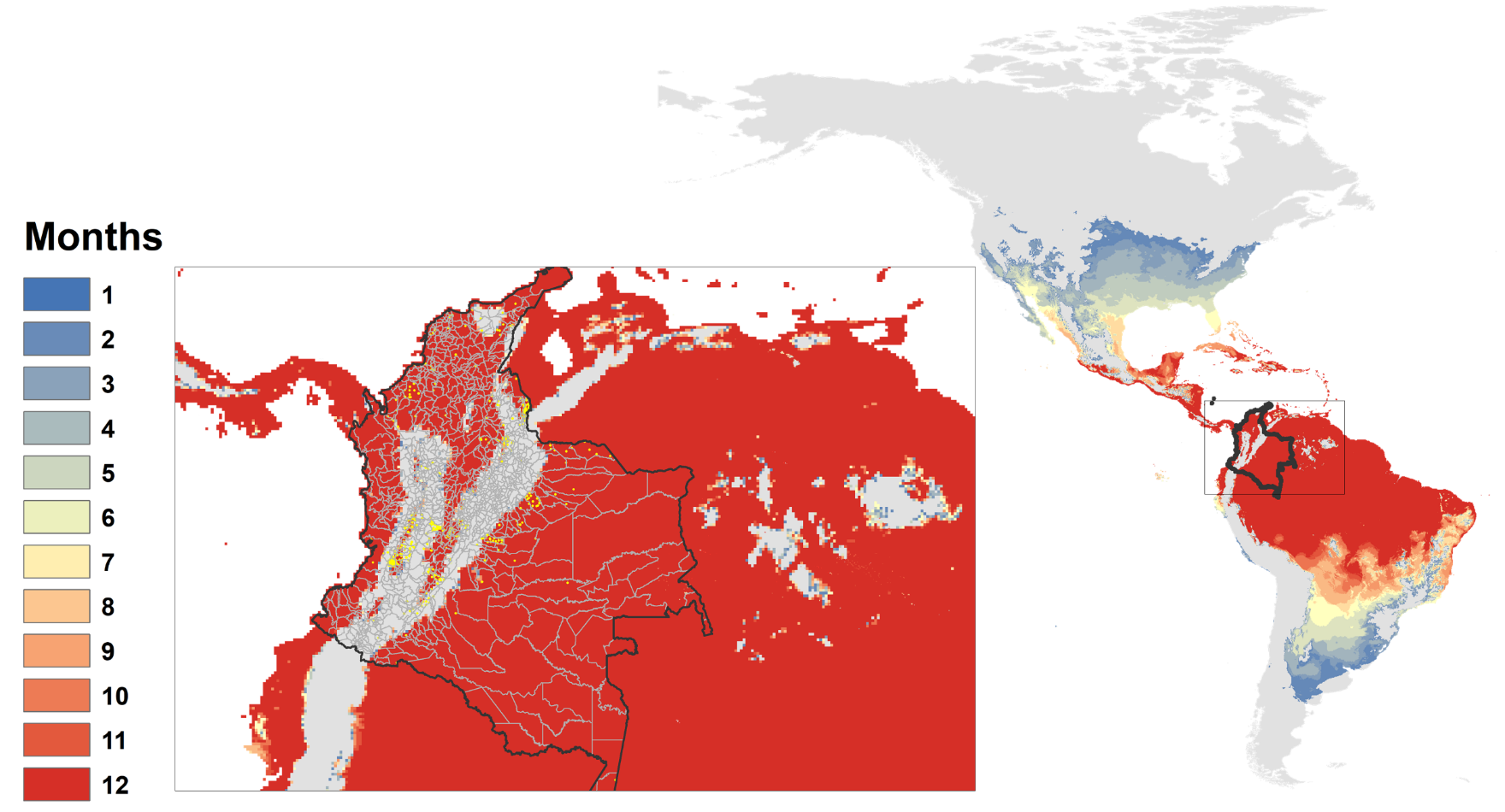
**

Spatial validation of the ZIKV suitability model, using case data from Colombia. Colombia is shown (black) in the Americas, on a backdrop of the ZIKV model mapped as months of suitability, described in Figure 5/6. Inset displays the case data at municipality level, dithered as dot densities (yellow, 300 cases per dot). Note contrasting suitability at the rapidly rising edge of the Andes, the most densely populated part of Colombia.

**References:**

1. Mordecai E., Cohen J., Evans M.V., Gudapati P., Johnson L.R., Lippi C.A., Miazgowicz K., Murdock C.C., Rohr J.R., Ryan S.J., et al. 2017 Detecting the impact of temperature on transmission of Zika, dengue, and chikungunya using mechanistic models. *PLoS Negl Trop Dis* **11**(4), e0005568. (doi:<https://doi.org/10.1371/journal.pntd.0005568>).
